# Complete genome sequence of *Sphaerobacter thermophilus* type strain (S 6022^T^)

**DOI:** 10.4056/sigs.601105

**Published:** 2010-01-28

**Authors:** Amrita Pati, Kurt LaButti, Rüdiger Pukall, Matt Nolan, Tijana Glavina Del Rio, Hope Tice, Jan-Fang Cheng, Susan Lucas, Feng Chen, Alex Copeland, Natalia Ivanova, Konstantinos Mavromatis, Natalia Mikhailova, Sam Pitluck, David Bruce, Lynne Goodwin, Miriam Land, Loren Hauser, Yun-Juan Chang, Cynthia D. Jeffries, Amy Chen, Krishna Palaniappan, Patrick Chain, Thomas Brettin, Johannes Sikorski, Manfred Rohde, Markus Göker, Jim Bristow, Jonathan A. Eisen, Victor Markowitz, Philip Hugenholtz, Nikos C. Kyrpides, Hans-Peter Klenk, Alla Lapidus

**Affiliations:** 1 DOE Joint Genome Institute, Walnut Creek, California, USA; 2 DSMZ – German Collection of Microorganisms and Cell Cultures GmbH, Braunschweig, Germany; 3 Los Alamos National Laboratory, Bioscience Division, Los Alamos, New Mexico, USA; 4 Oak Ridge National Laboratory, Oak Ridge, Tennessee, USA; 5 Biological Data Management and Technology Center, Lawrence Berkeley National Laboratory, Berkeley, California, USA; 6 HZI – Helmholtz Centre for Infection Research, Braunschweig, Germany; 7 University of California Davis Genome Center, Davis, California, USA

**Keywords:** *Sphaerobacteridae*, *Thermomicrobia*, thermophile, obligate aerobic, sewage sludge isolate, pleomorphic, non-motile, non-sporeforming, GEBA

## Abstract

*Sphaerobacter thermophilus* Demharter *et al*. 1989 is the sole and type species of the genus *Sphaerobacter*, which is the type genus of the family *Sphaerobacteraceae*, the order *Sphaerobacterales* and the subclass *Sphaerobacteridae*. Phylogenetically, it belongs to the genomically little studied class of the *Thermomicrobia* in the bacterial phylum *Chloroflexi*. Here, the genome of strain S 6022^T^ is described which is an obligate aerobe that was originally isolated from an aerated laboratory-scale fermentor that was pulse fed with municipal sewage sludge. We describe the features of this organism, together with the complete genome and annotation. This is the first complete genome sequence of the thermomicrobial subclass *Sphaerobacteridae*, and the second sequence from the chloroflexal class *Thermomicrobia*. The 3,993,764 bp genome with its 3,525 protein-coding and 57 RNA genes is a part of the *** G****enomic* *** E****ncyclopedia of* *** B****acteria and* *** A****rchaea * project.

## Introduction

Strain S 6022^T^ (DSM 20745 = ATCC 49802 = NCIMB 13125) is the type strain of the species *Sphaerobacter thermophilus*, representing the type species of the genus *Sphaerobacter*. *S. thermophilus* was described by Demharter *et al*. in 1989 [1]. It is Gram-positive, non-motile and non-sporeforming. It was originally isolated from thermal treated municipal sewage sludge from München-Grosslappen, Germany [2]. Cells of *S. thermophilus* were also identified in three other municipal sludge stabilization plants spread across Germany (Isenbüttel, Nettetal, and Gemmingen) using an immunolabelling procedure. From the operating parameters of these plants a minimum temperature growth range of 40-65°C can be predicted [2]. Here we present a summary classification and a set of features for *S. thermophilus* strain S 6022^T^, together with the description of the complete genomic sequencing and annotation.

## Classification and features

The closest related cultivated organism with a 16S rRNA sequence recorded in Genbank is *Thermomicrobium roseum* (DSM 5159) [3,4], which shares a mere 87% sequence similarity with strain S 6022^T^, indicating that *S. thermophilus* is phylogenetically one of the most isolated bacterial species. Only some uncultivated bacterial clones show a slightly closer relationship, e.g. clone Amb_16S_1237 (EF018775) isolated from *Populus tremula* (trembling aspen, 92%), EU035785 and EF643378 from soil in a radish-rich area in Jaunpur (India), clone AKYG1722 from farm soil adjacent to a silage storage bunker in Minnesota (89%), and AM935838 from a pilot-scale bioremediation process of hydrocarbon-contaminated soil in France (88%). None of the phylotypes sequenced during environmental screenings or genomic surveys surpassed 82% sequence similarity with strain S 6022^T^, expressly underlining the phylogenetically isolated and rare occurrence of *S. thermophilus* (status May 2009).

**Figure 1:**
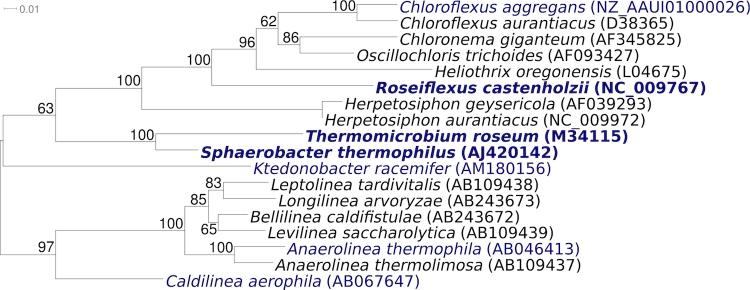
Phylogenetic tree of *S. thermophilus* strain S 6022^T^ and all type strains of the phylum *Chloroflexi*, inferred from 1,304 aligned characters [5,6] of the 16S rRNA gene sequence under the maximum likelihood criterion [7]. The tree was rooted with the members of *Anaerolineae* and *Caldilineae* within the *Chloroflexi*. The branches are scaled in terms of the expected number of substitutions per site. Numbers above branches are support values from 1,000 bootstrap replicates if larger than 60%. Lineages with type strain genome sequencing projects registered in GOLD [8] are shown in blue, published genomes in bold.

Figure 1 shows the phylogenetic neighborhood of *S. thermophilus* strain S 6022^T^ in a 16S rRNA based tree. The sequence of the sole 16S rRNA gene in the genome of strain S 6022^T^ differs by six nucleotides (0.4%) from the previously published 16S rRNA sequence generated from DSM 20745 (AJ420142). The difference between the genome data and the previously reported 16S rRNA in GenBank gene sequence is most likely due to sequencing errors in the latter.

**Figure 2:**
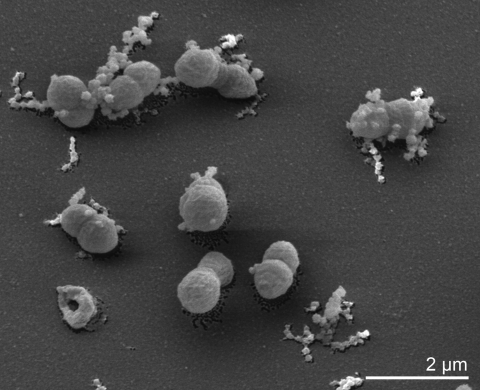
Scanning electron micrograph of *S. thermophilus* S strain 6022^T^

*S. thermophilus* S 6022^T^ cells are coccoid (Figure 2), but are also described as coccoid rods, 1-1.5 by 1.5-3 µm, in older cultures or in glucose-free medium irregular club- or dumb-bell shaped forms [1]. Branched cells are not observed. Colonies on Ottow Medium (DSMZ Medium No. 467) [9] are opaque, circular with entire margin and reach a diameter of 1-2 mm after 3 days of incubation at 60°C. The strain grows strictly aerobically with optimal growth at 55°C and pH 8.5 (Table 1). There is no acid production from glucose. Strain S 6022^T^ possesses catalase and oxidase and hydrolyzes starch but not gelatin, casein or cellulose [1]. Strain S 6022^T^ shares many features such as thermophilia, optimal pH for growth, and lack of motility with its closest relative, *T. roseum* (DSM 5159) [3,4]. The genome sequence as presented here might contribute to the solution of the question if *S. thermophilus*, like *T. roseum* encodes a complete flagellar system [4], although neither strain is motile. Interestingly, none of the other species in the *Chloroflexi* for which a genome sequence currently exists encode for any flagellar structural components [4].

**Table 1:** Classification and general features of *S. thermophilus* S 6022^T^ in accordance to the MIGS recommendations [10]

**MIGS ID**	**Property**	**Term**	**Evidence code**
	Current classification	Domain *Bacteria*	TAS [11]
		Phylum *Chloroflexi*	TAS [12]
		Class *Thermomicrobia*	TAS [11-14]
		Order *Sphaerobacterales*	TAS [15]
		Suborder *Sphaerobacterineae*	TAS [16]
		Family *Sphaerobacteraceae*	TAS [15]
		Genus *Sphaerobacter*	TAS [1]
		Species *Sphaerobacter thermophilus*	TAS [1]
		Type strain S 6022	
	Gram stain	positive	TAS [1]
	Cell shape	coccoid rods, irregular club- or dump-bell shaped	TAS [1]
	Motility	nonmotile	TAS [1]
	Sporulation	nonsporulating	TAS [1]
	Temperature range	thermophile, minimum 40-65°C	TAS [1,2]
	Optimum temperature	55°C, pH 8.5	TAS [1]
	Salinity	not reported	
MIGS-22	Oxygen requirement	obligate aerobic	TAS [1]
	Carbon source	starch	NAS
	Energy source	unknown	
MIGS-6	Habitat	thermal treated sewage sludge	TAS [2]
MIGS-15	Biotic relationship	free living	NAS
MIGS-14	Pathogenicity	none	NAS
	Biosafety level	1	TAS [17]
	Isolation	thermal treated sewage sludge	TAS [2]
MIGS-4	Geographic location	Munich, Germany	TAS [2]
MIGS-5	Sample collection time	between 1973 and 1988, probably 1984	TAS [1]
MIGS-4.1, MIGS-4.2	Latitude, Longitude	48.139, 11.58	NAS
MIGS-4.3	Depth	0 m	NAS
MIGS-4.4	Altitude	518 m	NAS

Evidence codes - IDA: Inferred from Direct Assay (first time in publication); TAS: Traceable Author Statement (i.e., a direct report exists in the literature); NAS: Non-traceable Author Statement (i.e., not directly observed for the living, isolated sample, but based on a generally accepted property for the species, or anecdotal evidence). These evidence codes are from of the Gene Ontology project [18]. If the evidence code is IDA, then the property was directly observed for a live isolate by one of the authors or an expert mentioned in the acknowledgements.

### Chemotaxonomy

Acid hydrolysates of the cell wall of strain S 6022^T^ yielded a ratio of glutamic acid to ornithine to alanine to β-alanine to muramic acid to glucosamine = 1:1.1:1.2:1.6:0.9:1.1. The murein structure type belongs to the murein variation A3β [19] with cross-linking via β-alanine [1]. The cell wall is unusually rich in protein content [1]. The principal isoprenoid quinone is an unsaturated menaquinone of type MK-8/0. MK-6/0, MK-7/0, MK-10/0 appear as minor constituents (4.8%, 7.7%, 12.8%) MK-6/0 [1]. Nothing is known about the spectrum of cellular fatty acids in the organism.

## Genome sequencing information

### Genome project history

This organism was selected for sequencing on the basis of its phylogenetic position, and is part of the *** G****enomic* *** E****ncyclopedia of* *** B****acteria and* *** A****rchaea * project. The genome project is deposited in the Genomes OnLine Database [8] and the complete genome sequence in GenBank. Sequencing, finishing and annotation were performed by the DOE Joint Genome Institute (JGI). A summary of the project information is shown in Table 2.

**Table 2:** Genome sequencing project information

**MIGS ID**	**Property**	**Term**
MIGS-13	Finishing quality	Finished
MIGS-28	Libraries used	Three genomic libraries: two Sanger libraries - 8 kb pMCL200 and fosmid pcc1Fos - and one 454 Pyrosequence standard library
MIGS-29	Sequencing platforms	ABI3730, 454 GS FLX, Illumina GA
MIGS-31.2	Sequencing coverage	7.4× Sanger; 28.5× Pyrosequence
MIGS-30	Assemblers	Newbler version 1.1.02.15, Arachne
MIGS-32	Gene calling method	Prodigal, GenePRIMP
	INSDC ID	CP001823 (chromosome), CP001824 (plasmid)
	GenBank date of release	November 23, 2009
	GOLD ID	Gc01151
	NCBI project ID	21087
	Database: IMG-GEBA	2502082099
MIGS-13	Source material identifier	DSM 20745
	Project relevance	Tree of Life, GEBA

### Growth conditions and DNA isolation

*S. thermophilus* S 6022^T^, DSM 20745, was grown in DSMZ medium 467 [20] at 55°C. DNA was isolated from 1-1.5 g of cell paste using Qiagen Genomic 500 DNA Kit (Qiagen, Hilden, Germany) following the manufacturer's instructions with modification st/FT for cell lysis according to Wu *et al*. [21].

### Genome sequencing and assembly

The genome was sequenced using a combination of Sanger, 454 and Illumina sequencing platforms. All general aspects of library construction and sequencing performed at the JGI can be found at http://www.jgi.doe.gov/. 454 Pyrosequencing reads were assembled using the Newbler assembler version 1.1.02.15 (Roche). Large Newbler contigs were broken into 4,435 overlapping fragments of 1,000 bp and entered into the assembly as pseudo-reads. The sequences were assigned quality scores based on Newbler consensus q-scores with modifications to account for overlap redundancy and to adjust inflated q-scores. A hybrid 454/Sanger assembly was made using the Arachne assembler. Possible mis-assemblies were corrected and gaps between contigs were closed by custom primer walks from sub-clones or PCR products. A total of 109 Sanger finishing reads were produced. Illumina reads were used to improve the final consensus quality using an in-house developed tool (the Polisher – publication in preparation). The final assembly consists of 35,091 Sanger and 516,954 Roche/454 reads. The error rate of the completed genome sequence is less than 1 in 100,000. Together all sequence types provided 35.9× coverage of the genome.

### Genome annotation

Genes were identified using Prodigal [22] as part of the Oak Ridge National Laboratory genome annotation pipeline, followed by a round of manual curation using the JGI GenePRIMP pipeline (http://geneprimp.jgi-psf.org/) [23]. The predicted CDSs were translated and used to search the National Center for Biotechnology Information (NCBI) nonredundant database, UniProt, TIGRFam, Pfam, PRIAM, KEGG, COG, and InterPro databases. Additional gene prediction analysis and functional annotation was performed within the Integrated Microbial Genomes - Expert Review (http://img.jgi.doe.gov/er) platform [24].

## Genome properties

**Figure 3:**
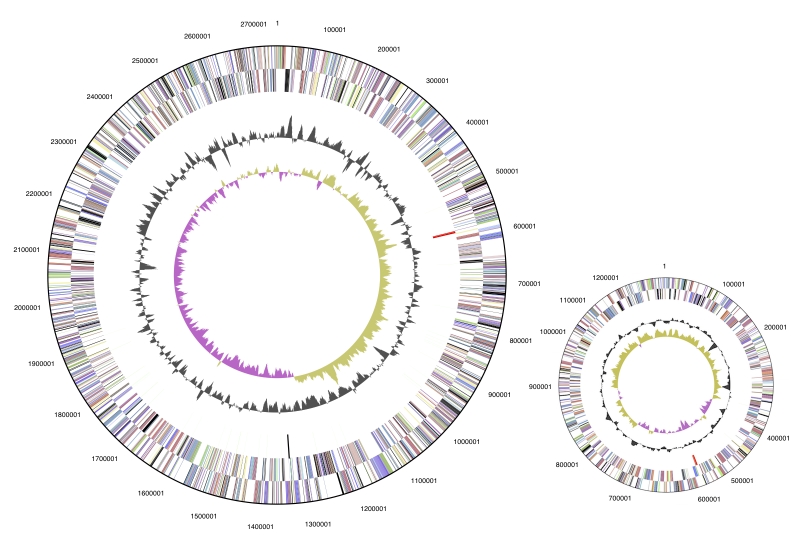
Graphical circular map of the genome. Chromosome (left), plasmid (right), drown not in scale. From outside to the center: Genes on forward strand (color by COG categories), Genes on reverse strand (color by COG categories), RNA genes (tRNAs green, rRNAs red, other RNAs black), GC content, GC skew.

The two replicons containing genome is 3,993,764 bp long with a 68.1% GC content (Table 3 and Figure 3). Of the 3,582 genes predicted, 3525 were protein coding genes, and 57 RNAs; 40 pseudogenes were also identified. The majority of the protein-coding genes (72.3%) were assigned a putative function while those remaining were annotated as hypothetical proteins. The properties and the statistics of the genome are summarized in Table 4.

**Table 3:** Genome Statistics

**Attribute**	**Value**	**% of Total**
Genome size (bp)	3,993,764	100.00%
DNA coding region (bp)	3,461,586	86.67%
DNA G+C content (bp)	2,720,128	68.11%
Number of replicons	2	
Extrachromosomal elements	0	
Total genes	3,582	100.00%
RNA genes	57	1.59%
rRNA operons	2	
Protein-coding genes	3,525	98.41%
Pseudo genes	40	1.12%
Genes with function prediction	2,591	72.33%
Genes in paralog clusters	677	18.90%
Genes assigned to COGs	2,619	73.12%
Genes assigned Pfam domains	2,679	74.79%
Genes with signal peptides	709	19.79%
Genes with transmembrane helices	908	25.35%
CRISPR repeats	1	

**Table 4:** Number of genes associated with the general COG functional categories

**Code**	**value**	**%age **	**Description**
J	162	4.6	Translation, ribosomal structure and biogenesis
A	0	0.0	RNA processing and modification
K	162	4.6	Transcription
L	121	3.4	Replication, recombination and repair
B	2	0.1	Chromatin structure and dynamics
D	26	0.7	Cell cycle control, mitosis and meiosis
Y	0	0.0	Nuclear structure
V	61	1.7	Defense mechanisms
T	109	3.1	Signal transduction mechanisms
M	173	4.9	Cell wall/membrane biogenesis
N	36	1.0	Cell motility
Z	0	0.0	Cytoskeleton
W	0	0.0	Extracellular structures
U	47	1.3	Intracellular trafficking and secretion
O	108	3.1	Posttranslational modification, protein turnover, chaperones
C	226	6.4	Energy production and conversion
G	157	4.5	Carbohydrate transport and metabolism
E	404	11.5	Amino acid transport and metabolism
F	60	1.7	Nucleotide transport and metabolism
H	145	4.1	Coenzyme transport and metabolism
I	111	3.1	Lipid transport and metabolism
P	163	4.6	Inorganic ion transport and metabolism
Q	79	2.2	Secondary metabolites biosynthesis, transport and catabolism
R	373	10.6	General function prediction only
S	207	5.9	Function unknown
-	963	27.3	Not in COGs
